# Supplementary material for: NEIL1 and NEIL2 DNA glycosylases modulate anxiety and learning in a cooperative manner in mice
Source: Commun Biol. 2021 Dec 2;4:1354. doi: 10.1038/s42003-021-02864-x (PMC8639745; doi:10.1038/s42003-021-02864-x)
Supplement: Supplementary file 2 — Supplementray Information [file 42003_2021_2864_MOESM2_ESM.pdf]

SUPPLEMENTARY INFORMATION

NEIL1 and NEIL2 DNA glycosylases modulate anxiety and learning in a cooperative manner in mice

Gunn A. Hildrestrand[1,†], Veslemøy Rolseth[1,2,†], Nicolas Kunath[3,†], Rajikala Suganthan[1], Vidar Jensen[4], Anna M. Bugaj[3], Marion S. Fernandez-Berrocal[3], Sunniva B. Sikko[3], Susanne Vetlesen[1], Anna Kuśnierczyk[5], Ann-Karin Olsen[6,7], Kristine B. Gützkow[6,7], Alexander D. Rowe[1,8], Wei Wang[3], Olve Moldestad[9,10], Monica D. Syrstad[1], Geir Slupphaug[5], Lars Eide[11], Arne Klungland[1,12], Pål Sætrom[5], Luisa Luna[1], Jing Ye[3], Katja Scheffler[3,13,14,§] and Magnar Bjørås[1,3,§,*]

[1]Department of Microbiology, Oslo University Hospital and University of Oslo, N-0424 Oslo, Norway
[2]Present address: Department of Forensic Sciences, Division of Laboratory Medicine, Oslo University Hospital, N-0424 Oslo, Norway
[3]Department of Clinical and Molecular Medicine, Norwegian University of Science and Technology, N-7491 Trondheim, Norway
[4]GliaLab, Department of Molecular Medicine, Institute of Basic Medical Sciences, University of Oslo, N-0317 Oslo, Norway
[5]Proteomics and Modomics Experimental Core Facility, Norwegian University of Science and Technology, N-7491 Trondheim, Norway
[6]Department of Molecular Biology, National Institute of Public Health, N-0456 Oslo, Norway
[7]Centre of Excellence "Centre for Environmental Radiation" (CERAD), NMBU, N-1433 Ås, Norway
[8]Present address: Department of Newborn Screening, Division of Paediatric and Adolescent Medicine, Oslo University Hospital, N-0424 Oslo, Norway
[9]Laboratory of Cellular Neurophysiology and Ion Channel Function, Department of Molecular Medicine, Institute of Basic Medical Sciences, University of Oslo, N-0317 Oslo, Norway
[10]Present address: Department for Rare Disorders, Division of Paediatric and Adolescent Medicine, Oslo University Hospital, N-0424 Oslo, Norway
[11]Department of Medical Biochemistry, Oslo University Hospital and University of Oslo, N-0424 Oslo, Norway
[12]Present address: Department of Biosciences, University of Oslo, N-0371 Oslo, Norway
[13]Department of Neuromedicine and Movement Science, Norwegian University of Science and Technology, N-7491 Trondheim, Norway
[14]Department of Neurology, St. Olavs Hospital, N-7006 Trondheim, Norway

* To whom correspondence should be addressed (lead contact). Tel: +47 95081497; Email: magnar.bjoras@ntnu.no
† These authors contributed equally.
§ These authors jointly supervised this work.

Supplementary Figure 1

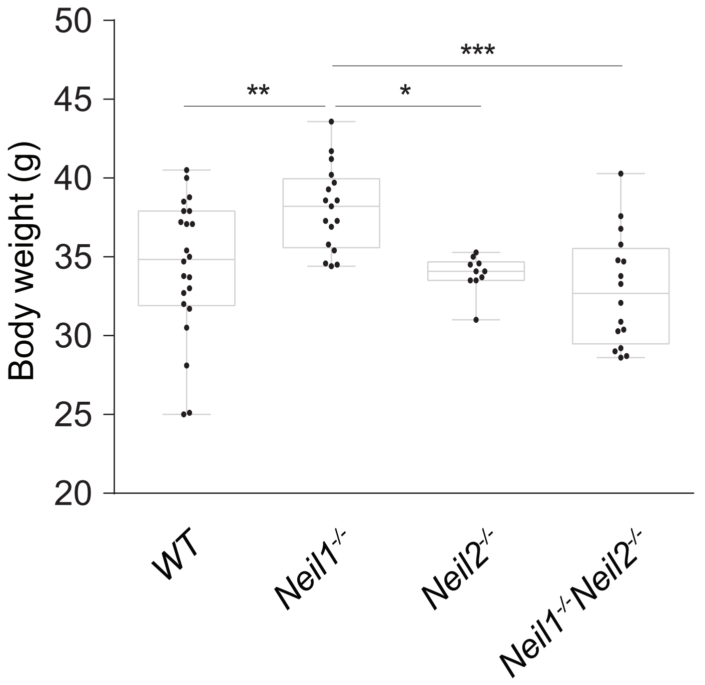

Supplementary Figure 1. Increased body weight in NEIL1-deficient mice.

The mice were weighed after the behavioral studies were completed. Data are shown in full, with overlaid boxplots representing the medians and interquartile ranges (IQR), Whiskers indicate min/max values. n = 22 WT, 17 *Neil1*$^{-/-}$, 10 *Neil2*$^{-/-}$ and 16 *Neil1*$^{-/-}$*Neil2*$^{-/-}$ mice. * $p < 0.0185$, ** $p < 0.0073$, *** $p < 0.0003$ by one-way ANOVA/Tukey.

Supplementary Figure 2

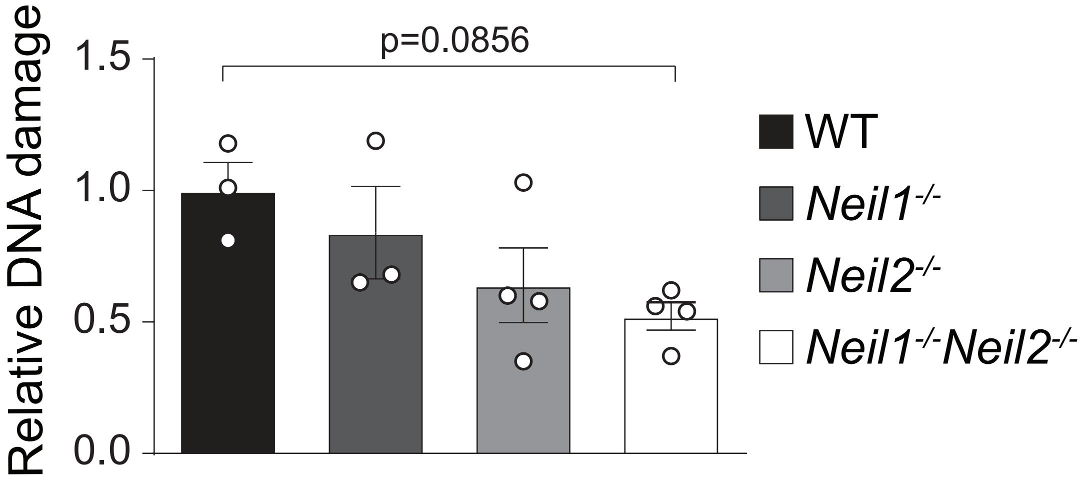

Supplementary Figure 2. Tendency to reduced DNA damage level in NEIL1/NEIL2 -deficient mice. Hippocampal DNA damage was measured by using a restriction enzyme-based qPCR method detecting damage in the Gapdh gene. Data are shown as mean + SEM. n = 3 WT, 3 *Neil1*$^{-/-}$, 4 *Neil2*$^{-/-}$ and 4 *Neil1*$^{-/-}$*Neil2*$^{-/-}$ mice. p-value by one-way ANOVA/Tukey.

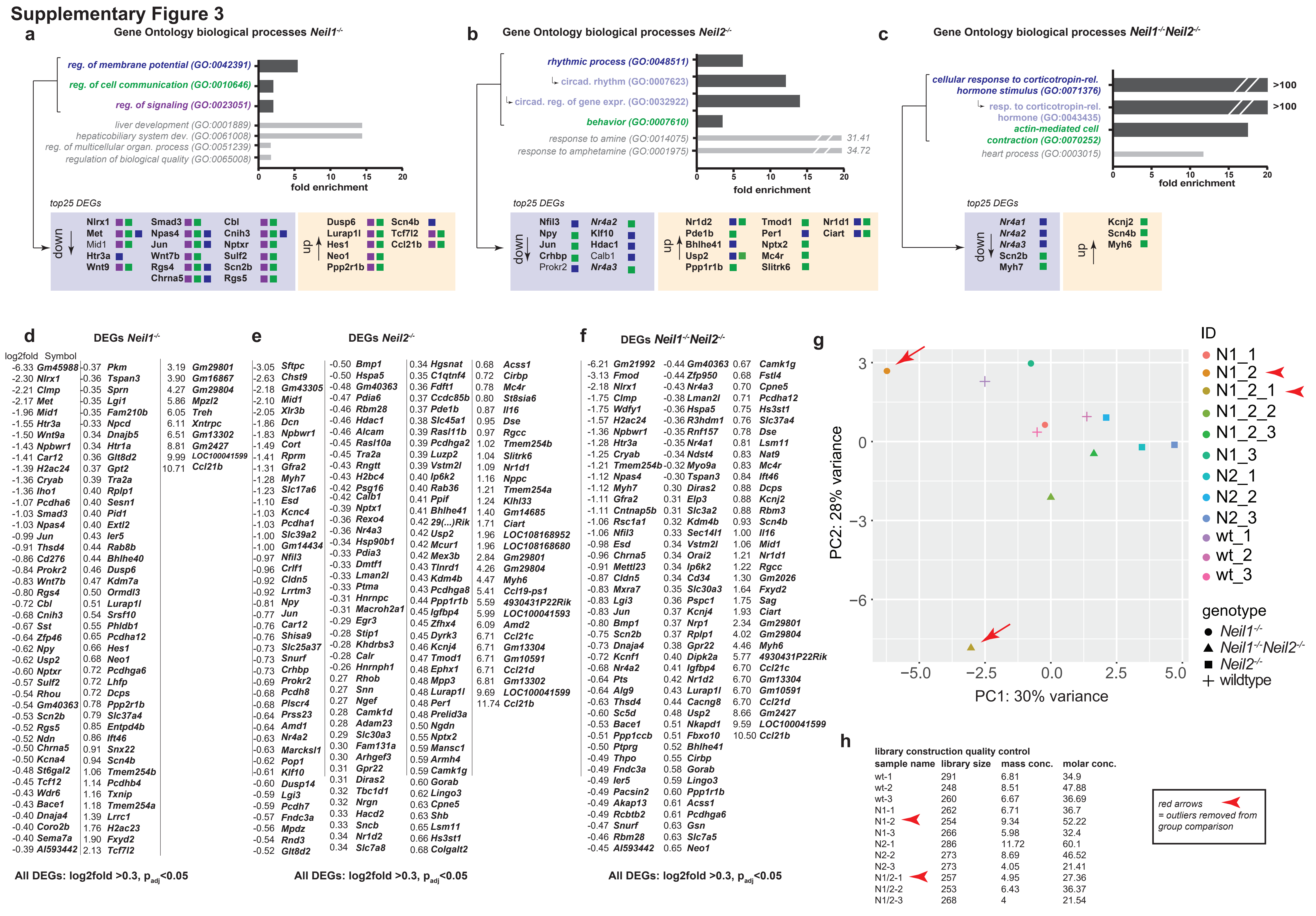

Supplementary Figure 3. NEIL1 and NEIL2 deficiency have a differential effect on CA1 gene expression. *(a-c)* GO-terms relevant for CNS function in all three genotypes (PANTHER Calssification System release 15.0, analysis 8/2020, FDR<0.05, fold enrichment >2). *(d-f)* All DEGs for all genotypes (log2fold >0.3, padj <0.05). *(g)* Exploratory data analysis revealed two outliers, which were subsequently excluded from the group comparison (red arrows, see also PCA plot without outliers in suppl.fig.4). Individual color dots indicate individual animals. *(h)* Library contsruction quality control, outliers marked with red arrows.

Supplementary Figure 4

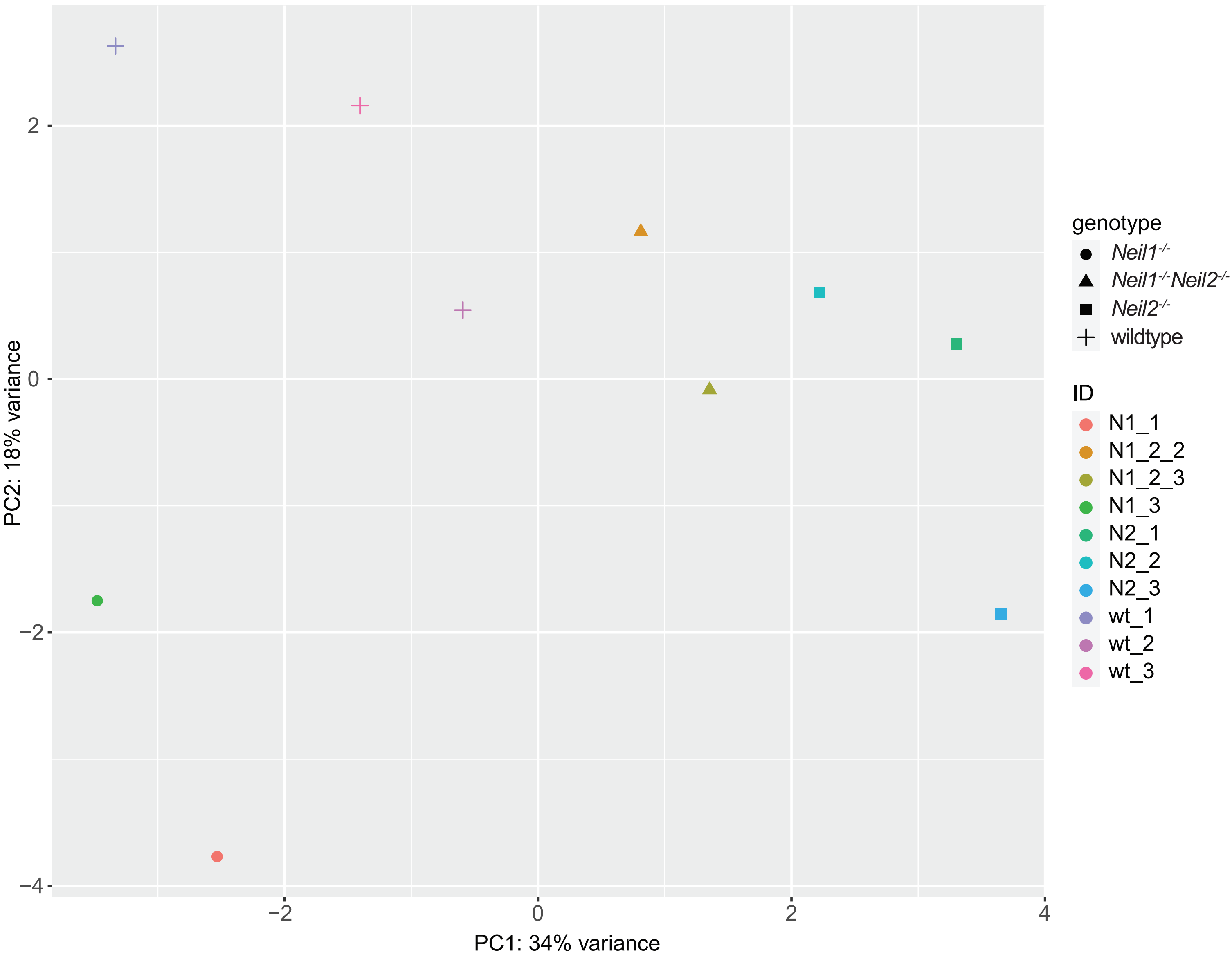

Supplementary Figure 4: Addendum to *Fig. S3, g*: PCA plot without outliers; individual color dots indicate individual animals

Supplementary Figure 5

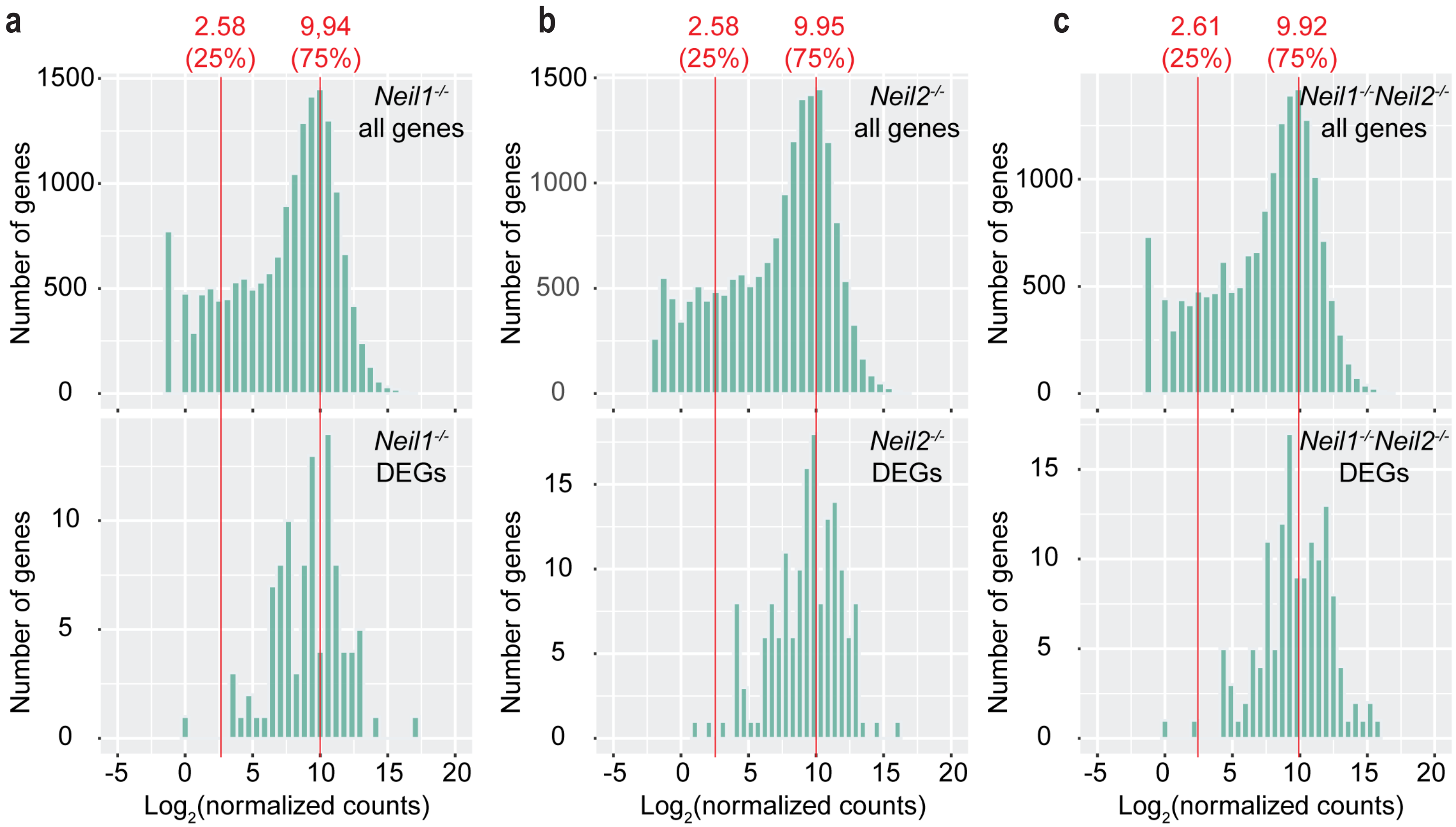

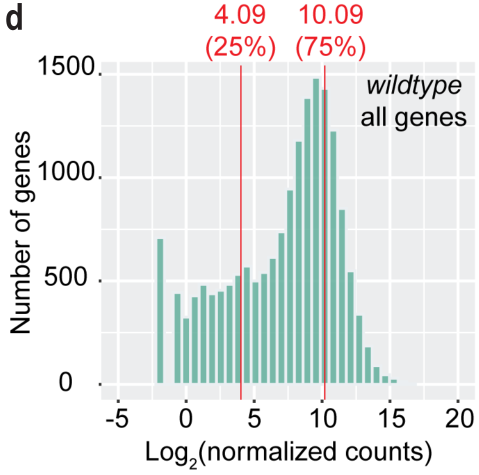

Supplementary Figure 5: NEIL-deficient mice alter preferentially high-expressed genes. Normalized counts (in log2 scale) of all sequenced genes (upper panel) and the DEGs (lower panel) in *Neil1*$^{-/-}$ (a), *Neil2*$^{-/-}$ (b), *Neil1*$^{-/-}$*Neil2*$^{-/-}$ (c) and wildtype (d) mice were shown in histograms. The 25% lower quartile and 75% upper quartile (based on all sequenced genes, indicated by the red line) were used as criteria to define the low- and high-expressed genes, respectively.

Supplementary Figure 6

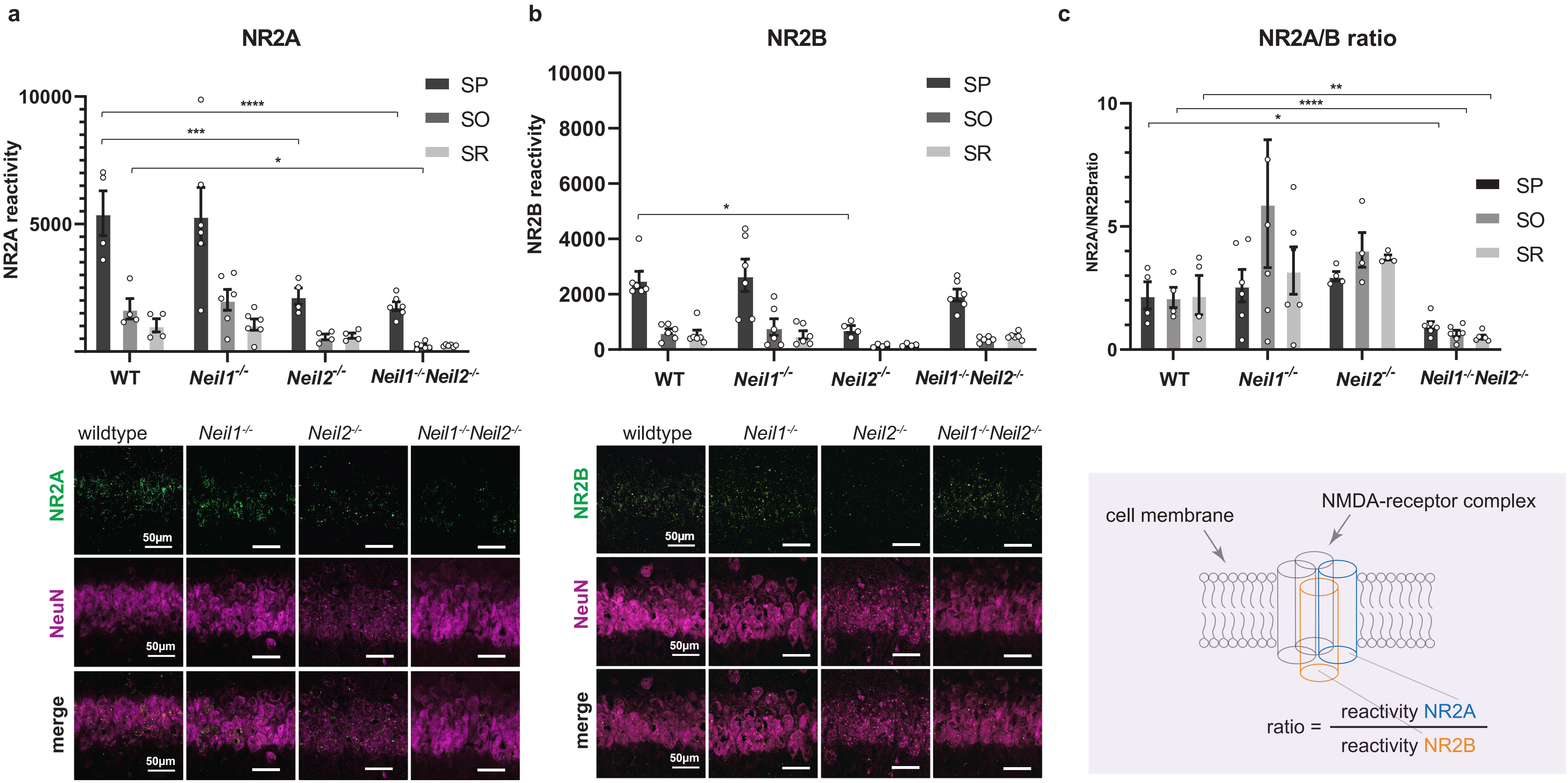

Supplementary Figure 6. Altered composition of regulatory NMDA-receptor subunits in *Neil1*$^{-/-}$*Neil2*$^{-/-}$ CA1. Immunoreactivity levels of (a) NR2A and (b) NR2B and (c) NR2A vs. NR2B ratio in stratum pyramidale (SP), stratum oriens (SO) and stratum radiatum (SR) of CA1. Representative images (centered over SP) and an illustration of the NMDA-receptor complex are shown. Data are presented as “reactivity levels” based on Imaris spot detection tool (a) and (b) and as ratio of “reactivity levels” (c) + SEM. n = 6 slices (3 animals, 2 slices per animal), statistics conducted at animal level (see methods) per genotype. (a) * $p = 0.0323$, *** $p = 0.0003$ and **** $p < 0.0001$ and (B) * $p = 0.0354$ by two-way ANOVA/Tukey. (C) * $p = 0.01$, ** $p = 0.001$, **** $p < 0.0001$ by multiple t-tests with Holms-Sidak-correction.

Supplementary Figure 7

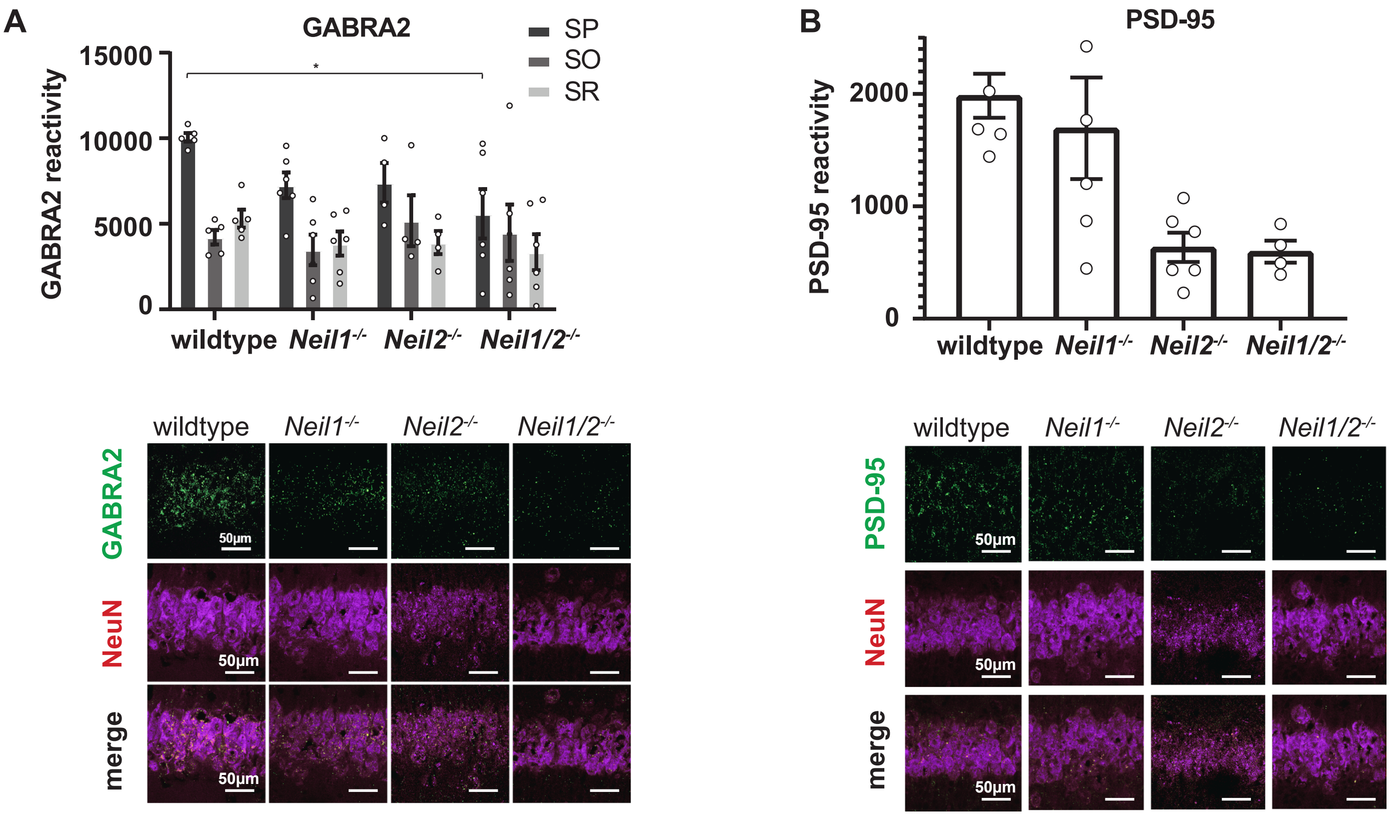

Supplementary Figure 7. Reduced expression of GABRA in CA1. (A) GABA-A-receptor α2 subunit (GABRA2) expression across CA1 stratum pyramidale (SP), stratum oriens (SO) and stratum radiatum (SR). * $p = 0.0314$ by two-way ANOVA/Tukey ($p = 0.1369$ for *Neil2*$^{-/-}$ in SP). (B) Expression of PSD-95 across SP (SR and SO were not examined due to bad immunostaining signal; $p = 0.0762$ and 0.0983 for *Neil2*$^{-/-}$ and *Neil1*$^{-/-}$*Neil2*$^{-/-}$, respectively, by one-way-ANOVA/Tukey). (A and B) Data are presented as “reactivity levels” based on Imaris spot detection tool ± SEM. n = 6 animals (2 slices each), statistics at animal level (see methods).
