# Supplementary material for: NEIL1 and NEIL2 DNA glycosylases modulate anxiety and learning in a cooperative manner in mice
Source: Commun Biol. 2021 Dec 2;4:1354. doi: 10.1038/s42003-021-02864-x (PMC8639745; doi:10.1038/s42003-021-02864-x)
Supplement: Supplementary file 3 — Description of additional supplementary files [file 42003_2021_2864_MOESM3_ESM.pdf]

Description of Additional Supplementary Files

File name: Supplementary Data 1

Description: Figure source data (Fig. 1, 2b-c, 3 and Supplementary Figures 1, 2, 6 and 7)
