# Supplementary material for: NEIL1 and NEIL2 DNA glycosylases modulate anxiety and learning in a cooperative manner in mice
Source: Commun Biol. 2021 Dec 2;4:1354. doi: 10.1038/s42003-021-02864-x (PMC8639745; doi:10.1038/s42003-021-02864-x)
Supplement: Supplementary file 5 — Reporting Summary [file 42003_2021_2864_MOESM5_ESM.pdf]

nature research

Corresponding author(s): Magnar Bjørås

Last updated by author(s): Oct 20, 2021

Reporting Summary

Nature Research wishes to improve the reproducibility of the work that we publish. This form provides structure for consistency and transparency in reporting. For further information on Nature Research policies, see our Editorial Policies and the Editorial Policy Checklist.

Statistics

For all statistical analyses, confirm that the following items are present in the figure legend, table legend, main text, or Methods section.

n/a	Confirmed	
☐	☒	The exact sample size (*n*) for each experimental group/condition, given as a discrete number and unit of measurement
☐	☒	A statement on whether measurements were taken from distinct samples or whether the same sample was measured repeatedly
☐	☒	The statistical test(s) used AND whether they are one- or two-sided<br>*Only common tests should be described solely by name; describe more complex techniques in the Methods section.*
☒	☐	A description of all covariates tested
☐	☒	A description of any assumptions or corrections, such as tests of normality and adjustment for multiple comparisons
☐	☒	A full description of the statistical parameters including central tendency (e.g. means) or other basic estimates (e.g. regression coefficient) AND variation (e.g. standard deviation) or associated estimates of uncertainty (e.g. confidence intervals)
☒	☐	For null hypothesis testing, the test statistic (e.g. F, t, r) with confidence intervals, effect sizes, degrees of freedom and P value noted<br>*Give P values as exact values whenever suitable.*
☒	☐	For Bayesian analysis, information on the choice of priors and Markov chain Monte Carlo settings
☒	☐	For hierarchical and complex designs, identification of the appropriate level for tests and full reporting of outcomes
☒	☐	Estimates of effect sizes (e.g. Cohen's *d*, Pearson's *r*), indicating how they were calculated

Our web collection on statistics for biologists contains articles on many of the points above.

Software and code

Policy information about availability of computer code

Data collection	All software used to collect data are described in the Methods section of the manuscript.
Data analysis	All software used to analyse data are described in the Methods section of the manuscript.

For manuscripts utilizing custom algorithms or software that are central to the research but not yet described in published literature, software must be made available to editors and reviewers. We strongly encourage code deposition in a community repository (e.g. GitHub). See the Nature Research guidelines for submitting code & software for further information.

Data

Policy information about availability of data

All manuscripts must include a data availability statement. This statement should provide the following information, where applicable:

- Accession codes, unique identifiers, or web links for publicly available datasets
- A list of figures that have associated raw data
- A description of any restrictions on data availability

The RNA sequence data generated and analyzed during the current study have been deposited in NCBI's Gene Expression Ominbus, accession number GSE160621 (https://www.ncbi.nlm.nih.gov/geo/query/acc.cgi?acc=GSE160621). The DNA sequence data have been deposited in European Nucleotide Archive (ENA), accession number PRJEB31108 (https://www.ebi.ac.uk/ena/browser/view/PRJEB31108?show=reads). The figure source data are available in Supplementary Data 1. Further information and requests for resources and reagents should be directed to and will be fulfilled by the Lead Contact, Magnar Bjørås (magnar.bjoras@ntnu.no).

Field-specific reporting

Please select the one below that is the best fit for your research. If you are not sure, read the appropriate sections before making your selection.

[x] Life sciences   [ ] Behavioural & social sciences   [ ] Ecological, evolutionary & environmental sciences

For a reference copy of the document with all sections, see nature.com/documents/nr-reporting-summary-flat.pdf

Life sciences study design

All studies must disclose on these points even when the disclosure is negative.

Sample size	Sample size was determined based on previous studies in our lab.
Data exclusions	No data were excluded from the analyses.
Replication	Behavioral studies are time consuming and in stead of repeating the experiments, reproducibility was assured by including more animals in one study. For all other experiments attempts of replication were successful.
Randomization	Allocation into groups was determined by the genotypes of the mice.
Blinding	The investigators were not blinded to group allocation during data collection and/or data analysis.

Reporting for specific materials, systems and methods

We require information from authors about some types of materials, experimental systems and methods used in many studies. Here, indicate whether each material, system or method listed is relevant to your study. If you are not sure if a list item applies to your research, read the appropriate section before selecting a response.

Materials & experimental systems

n/a	Involved in the study	
[ ]	[x]	Antibodies
[x]	[ ]	Eukaryotic cell lines
[x]	[ ]	Palaeontology and archaeology
[ ]	[x]	Animals and other organisms
[x]	[ ]	Human research participants
[x]	[ ]	Clinical data
[x]	[ ]	Dual use research of concern

Methods

n/a	Involved in the study	
[x]	[ ]	ChIP-seq
[x]	[ ]	Flow cytometry
[x]	[ ]	MRI-based neuroimaging

Antibodies

Antibodies used	Primary antibodies: GABRA2 rabbit polyclonal, Cat.No.224103, Synaptic Systems, Goettingen, Germany); NR2A rabbit polyclonal ,Cat.No. AGC-002, alomone labs, Jerusalem, Israel; NR2B rabbit polyclonal, Cat.No. AGC-003, alomone labs, Jerusalem, Israel; NeuN mouse monoclonal, A40/MAB377, Cat.No. 636574, ThermoFisher, Waltham, MA, USA; PSD-95 rabbit polyclonal, Cat.No. PA585769, ThermoFisher, Waltham, MA, USA. Secondary antibodies: Alexa Flour dyes (A488, A555, A647), Thermo Fisher Scientific, Waltham, MA, USA.
Validation	All antibodies used are commercial and validation may be obtained from the vendor's web site.

Animals and other organisms

Policy information about studies involving animals; ARRIVE guidelines recommended for reporting animal research

Laboratory animals	Mus musculus, C57BL/6N, males, aged 3-6 months, were used.
Wild animals	The study did not involve wild animals.
Field-collected samples	The study did not involve samples collected from the field.
Ethics oversight	All experiments were approved by the Norwegian Animal Research Authority and conducted in accordance with the laws and regulations controlling experimental procedures in live animals in Norway and the European Union’s Directive 2010/63/EU.

Note that full information on the approval of the study protocol must also be provided in the manuscript.
